# Targeting self-renewal pathways in myeloid malignancies

**DOI:** 10.1186/1478-811X-11-33

**Published:** 2013-05-15

**Authors:** William A Sands, Mhairi Copland, Helen Wheadon

**Affiliations:** 1 Paul O’Gorman Leukaemia Research Centre, College of Medical, Veterinary and Life Sciences, University of Glasgow, Gartnavel General Hospital, 1053 Great Western Road, Glasgow G12 0ZD, UK

**Keywords:** Myeloid, Leukemia, Self-renewal, Cross-talk, Therapy, Hematopoietic stem cell

## Abstract

A fundamental property of hematopoietic stem cells (HSCs) is the ability to self-renew. This is a complex process involving multiple signal transduction cascades which control the fine balance between self-renewal and differentiation through transcriptional networks. Key activators/regulators of self-renewal include chemokines, cytokines and morphogens which are expressed in the bone marrow niche, either in a paracrine or autocrine fashion, and modulate stem cell behaviour. Increasing evidence suggests that the downstream signaling pathways induced by these ligands converge at multiple levels providing a degree of redundancy in steady state hematopoiesis. Here we will focus on how these pathways cross-talk to regulate HSC self-renewal highlighting potential therapeutic windows which could be targeted to prevent leukemic stem cell self-renewal in myeloid malignancies.

## Lay abstract

Leukemia is often a stem cell disease. Here we describe mechanisms that keep diseased cells in a stem like state and how they are being exploited to treat certain leukemias.

## Steady-state hematopoiesis

In adults hematopoiesis occurs within the architecture of the bone marrow (BM), a specialized microenvironment referred to as the stem cell niche where the hematopoietic stem cells (HSCs) reside and are regulated for quiescence, self-renewal and differentiation through intrinsic and extrinsic mechanisms. The BM contains at least two distinctive HSC supportive niches: an endosteal osteoblastic niche, which supports quiescence and self-renewal and a more vascular/peri-sinusoidal niche that promotes proliferation and differentiation [1]. Within the more hypoxic osteoblastic niche, HSCs specifically interact via N-cadherin and Jagged 1 with the osteoblasts that line the endosteal surface. The osteoblasts secrete several factors including; stem cell factor (SCF), thrombopoietin (TPO), angiopoietin-1, osteopontin, Wnt, and CXC motif ligand 12 (CXCL12; also termed stromal-derived factor-1 [SDF-1]) which are important regulators of quiescence and HSC maintenance [2]. *In vivo* imaging indicates that the HSCs and progenitors located further away from this area, in the vicinity of sinusoidal endothelial cells near the vascular endosteum, are more proliferative [3]. In these BM areas, HSCs interact with endothelial cells via specific cell adhesion molecules; E-selectin, P-selectin, VCAM1 and ICAM1. Endothelial cells secrete several factors important for HSC homeostasis including CXCL12, vascular endothelial growth factor (VEGF), transforming growth factor β (TGFβ), fibroblast growth factor 4 (FGF4), adrenomedullin, insulin-like growth factor binding protein 2 (IGFB2), angiopoietin-like protein 5 (Angptl-5) and pleiotrophin. E-selectin and CXCL12 are important for BM homing of circulating HSCs, whereas VEGF plays a role in controlling HSC self-renewal and repopulating ability, and TGF−β is known to inhibit HSC proliferation, promoting HSC quiescence. Recent evidence indicates that IGFB2 and Angptl-5 are involved in HSC expansion with pleiotrophin, CXCL12 and FGF4 all mediating HSC progenitor interactions with the vascular niche and facilitating differentiation [2].

Myeloid leukemia occurs due to genetic changes in an HSC, or in some cases, a committed progenitor, that then acquires self-renewing properties, and thus the ability to successively transfer leukemia by adoptive horizontal transfer to recipients [4,5]. This gives rise to a hierarchical clonal stem cell disease often with more than one expanding leukemic stem cell (LSC) population sustaining the malignancy, with the BM being the primary site of disease development. In acute myeloid leukemia (AML) CD34+ LSCs have been shown to be more akin to normal progenitors than CD34+ HSC with two populations identified as having engraftment potential in >80% of patients analysed. These consisted of a CD34^+^CD38^-^CD45RA^+^ lymphoid-primed multipotent progenitor (LMPP) population and a CD34^+^CD38^+^CD45RA^+^ granulocyte-macrophage progenitors (GMP) population [4]. Similar results were previously demonstrated when *MLL-ENL* was expressed in HSC with the efficiency of transplantation being HSC> common myeloid progenitor (CMP) >GMP, indicating that the more primitive populations required fewer cells for transformation with more committed progenitors still retaining this capacity [5]. Gene profiling also revealed that although the more mature LSC maintained normal GMP progenitor identity they had re-activated self-renewal programmes [4,5]. LSCs therefore retain all the fundamental properties of HSCs and progenitors including; quiescence, self-renewal and differentiation potential. In addition LSCs behave like normal HSCs and progenitors associating with BM stromal cells and extracellular matrix proteins via cell adhesion molecules [6], and rely on CXCL12-mediated CXCR4 signaling for homing and mobilization within the BM [6,7]. Thus, many of the molecules that mediate the interaction between stem cells and the BM niche are utilised by both HSCs and LSCs, suggesting LSC behaviour is likely to be modulated by interactions and signals received within the BM microenvironment. Indeed, many molecular lesions in LSCs affect pathways that are activated in the niche environment. These include ligand independent activating mutations of Flt3, cKit, MPL [8], and chromosomal translocations giving rise to constitutively active fusion proteins such as AML1/ETO in AML, PML/RARα in acute promyelocytic leukemia (APL), BCR/ABL in chronic myeloid leukemia (CML) [9], and the FGFR1 and PDGFR fusion partners which include ZNF198-FGFR1, FIPILI-PDGFRα and ETV6-PDGFRβ involved in chronic eosinophilic leukemia (CEL), juvenille myelomonocytic leukemia (JMML) and chronic myelomonocytic leukemia (CMML) [10,11]. A common theme is the acquisition of self-renewal promoting activity through the ability of these receptors and fusion proteins to activate signal transduction especially the PI3 Kinase (discussed below) and JAK/STAT pathways. The molecular mechanisms still need further elaboration, but possible target genes include HIF2α and HoxA9, and the down regulation of C/EBPα [12,13]. Emerging evidence indicates that not only do the LSCs strongly rely on the BM niche for their self-renewal and proliferation, but they may also modify it to their advantage. Indeed, abnormalities have been observed within the leukemic BM including elevated SCF, CXCL12 and VEGF levels as well as increased acidity and hypoxia [14]. Recent studies indicate that alterations within the BM microenvironment might also be an initiating trigger to induce leukemogenic transformation of normal cells. This is often the case for patients with BM failure syndromes such as acquired and inherited aplastic anemia. These patients are at risk of developing clonal neoplasms including AML, myelodysplastic syndromes (MDS) and paroxysmal nocturnal hemaglobinuria (PHN). Over-production of apoptotic inducing cytokines by T cells triggers BM failure and has been shown to lead to transformation through clonal selection and adaption of resistant HSC which are able to survive in this modified BM microenvironment [15]. In mouse models targeted disruption of retinoblastoma protein or retinoic acid receptor in the BM microenvironment results in the development of myeloproliferative disorders [16]. Whereas conditional knockout of *dicer1* in osteoblastic precursors, results in myelodysplasia and the development of AML [17]. These findings highlight that the BM niche plays a key role in regulating stem cell function and hematopoiesis.

## Discovery and persistence of LSC

Several myeloid malignancies including AML, CML, CMML and APL are stem/progenitor cell diseases. Data indicates that although LSCs are relatively rare, they maintain the disease and are responsible for relapse following chemotherapeutic elimination of the leukemic cell bulk. Their continued presence is in part due to their ability to self-renew and evade differentiation [18]. In myeloid leukemias, the presence of LSCs was initially proposed following limiting dilution studies of patient peripheral blood, and assessing colony forming ability. The first *in vivo* demonstration was in AML, where only one cell in 250 000 could initiate disease in an immunocompromised SCID mouse transplantation model [19]. Through a variety of experiments involving SCID mouse transplantation, using patient cells FACS sorted based on surface marker expression, the stem cell population was found to comprise of a primitive population with a CD34^+^CD38^-^ phenotype. More recent findings, using improved murine engraftment models have revealed heterogeneity in AML LSC phenotypes, which include cells that are CD34^+^CD38^+^ or even CD34^-^[19]. Given the importance of LSC for the maintenance of myeloid malignancies, their frequent resistance to chemotherapy, and their heterogeneous nature, understanding the deregulation of self-renewal pathways utilised by LSCs is necessary to design essential therapeutics to eliminate myeloid malignancies. Intervention may involve targeting the LSC self-renewal pathways or promoting their differentiation.

## Self-renewal pathways

**Figure 1:**
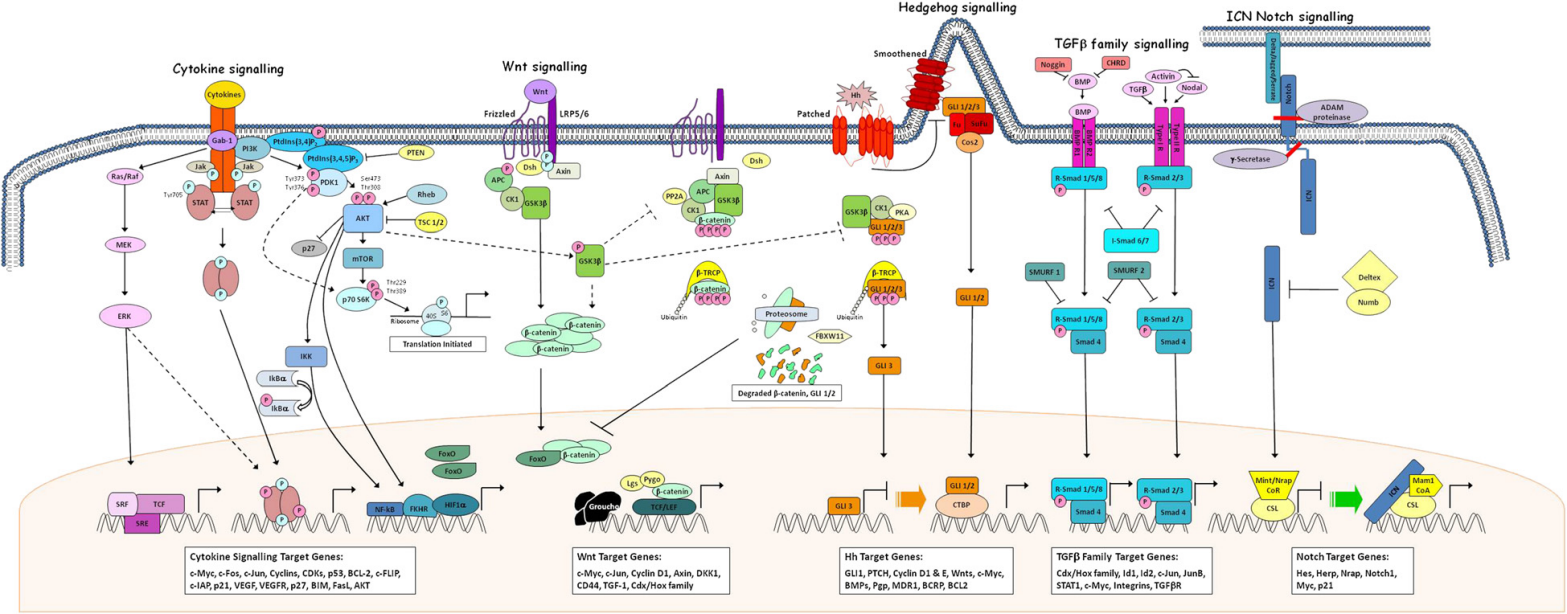
Cross-talk between self-renewal pathways involved in myeloid malignancies.

Although cytokines are known to regulate HSC functions *in vivo*, their ability to expand and maintain HSCs has been limited *in vitro*, with HSCs eventually differentiating and subsequently losing their reconstitution capacity. This is due to other factors augmenting HSC self-renewal within the BM niche, as summarized in Figure 1. Cytokines signal through several well-defined pathways including the Jak/STAT, Raf/MEK/ERK, NFkB and the PI3 Kinase/PTEN/Akt/mTOR signal transduction cascades. Cytokine independent activation of these pathways is a common feature in leukemia and has been shown to be an important mechanistic feature for survival and proliferation of the malignant clone.

## Phosphoinositide-3-kinase (PI3 Kinase) pathway

Evidence suggests that the PI3 Kinase pathway plays a critical role in both HSC and LSC self-renewal. This pathway leads to the activation of Akt via the Serine/Threonine protein kinase phosphoinositide-dependent kinase-1 (PDK1). Activated Akt phosphorylates numerous substrates to initiate several well described PI3 Kinase responses including; cell-cycle, stress resistance, survival, growth, metabolism, migration and long-term regeneration potential of HSCs. An important Akt substrate is glycogen synthase kinase 3β(GSK3β) which is inactivated by serine phosphorylation following PI3 Kinase signaling. Deregulation of this pathway can therefore potentiate Wnt and Hedgehog (Hh) signaling as GSK3β plays an important role in regulating β-catenin and Glioma (GLI) transcription factor levels (described below). Another Akt-dependant molecule is Protein Kinase A (PKA), which positively regulates Hh signaling through GLI inactivation [20]. Forkhead O (FoxO) transcription factors are effectors of the PI3 Kinase/Akt pathway and have been demonstrated to regulate HSC numbers and repopulation capacity in transgenic mouse models [21]. FoxOs may also play an important role in enhancing self-renewal and differentiation blockade via oxidative stress sensing. Reactive oxygen species (ROS) enhance FoxOs ability to bind and sequester β-catenin, preventing its degradation, thus enhancing canonical Wnt signaling [22]. The key negative regulator of PI3 Kinase signaling, often deleted or inactivated in cancers is the tumour suppressor phosphatase and tensin homolog (PTEN). Conditional knock-out studies using the Cre-loxP system to delete *Pten* in adult HSCs resulted in the mice developing transplantable AML and acute lymphoblastic leukemia (ALL), highlighting an important role for this pathway in leukemogenesis [23]. These mice however undergo HSC depletion. In situations where *Pten* is impaired, the problem of HSC depletion can be overcome with the use of the mTOR inhibitor rapamycin, which has the added advantage of depleting LSCs. This may be through reducing flux through the mTORC1 pathway, where in murine models at least, deletion of Raptor, a component of mTORC1, improves survival in Pten-deleted mice [24].

Despite the fact that many small molecule inhibitors of this pathway have been developed, there are currently few clinical trials in myeloid leukemias. However, one compound, the PI3 Kinase δ selective inhibitor CAL-101 is currently being tested in a phase 1 clinical trial with an extension study for patients with AML (Table 1) [25].

**Table 1:** Summary of available inhibitors which target self-renewal pathways involved in myeloid malignancies

**Pathway**	**Name of drug**	**Mode of action**	**Producer**	**Stage of clinical / non-clinical development**	**Trial number**
**Wnt/GSK3β**[26-32]	**Aspirin / NSAIDs**	Complex / chemoprevention	Generic	Over the counter medications; no ongoing clinical	
	**Vitamin A**	Reduce TCF-β-catenin	Generic	trials in haematological malignancies	
				Non-clinical	
	**Vitamin D**	complex formation	Leo	Non-clinical	
	**(EB1089)**	Binding of β-catenin by	Pharmaceuticals		
	**WNT1 and WNT2**	vitamin D receptor		Non-clinical	
	**McAbs**	Monoclonal antibodies	Generic		
	**XAV939**			Non-clinical	
	**ZTM000990,**	Inhibits tankyrase 1&2	Novartis	Non-clinical	
	**PKF118-310, PKF118-744, PKF222-815, CGP049090, PNU-74654 ICG-001 NSC668036 SB-216763**	Inhibit TCF-β-catenin	Multiple Institute for Chemical Genomics	Non-clinical Non-clinical	
			NCI	Non-clinical	
		complex			
		Inhibits β-catenin/CREB-binding protein transcription			
		Inhibits dishevelled PDZ domain			
		Inhibits GSK-3	Sigma		
	**GSK-3IX,**		Chemicals	Non-clinical	
		Inhibit GSK-3	EMD		
	**alsterpaullone**		Biosciences		
**Notch**[32,33]	**MK-0752**	γ-secretase inhibitor	Merck	Phase 1: T acute lymphoblastic leukemia	NCT00100152
	**GSI-I, GSI-IX, GSI-**	γ-secretase inhibitors	Calbiochem	Non-clinical	
	**X, GSI-XII, GSI-**	Monoclonal antibody	Generic	Non-clinical	
	**XXI**				
	**NOTCH3 McAb**				
**Hh**[34]	**Cyclopamine**	SMO antagonist	Generic	Non-clinical	
	**LDE225**	SMO antagonist	Novartis	Phase 1: CML – in combination with Nilotinib	NCT1456676
	**LEQ506**	SMO antagonist	Novartis	Solid tumours only (phase I & II)	
	**GDC-0449**	SMO antagonist	Genentech	Phase 1b: Myeloma in first remission or first relapse post ASCT	NCT01330173
	**BMS-833923**	SMO antagonist	Bristol-Myers Squibb	Phase 1: CML - in combination with Dasatinib	NCT01218477
				Phase 1b: Myeloma - in combination with Lenalidomide with Dexamethasone or Bortezomib with Dexamethasone	NCT00884546
	**IPI926**	SMO antagonist	Infinity	Phase 2: Myelofibrosis	NCT01371617
	**PF-04449913**	SMO antagonist	Pfizer	Phase 1: CML - in combination with Dasatinib or Bosutinib	NCT00953758
	**GANT61**	Direct GLI inhibition	Generic	Non-clinical	
**FOXO/TGFβ**[35]	**Rapamycin**	Inhibitor of mTOR	Generic	No current clinical trials in haematological malignancies	
	**LY294002**	PI3K inhibitor			
	**LY364947**	PI3K inhibitor	Merck	Non-clinical	
			Merck	Non-clinical	
**C-MYB**[36]	**C-MYB AS ODN**	Infusional C-MYB anti-sense oligodeoxy nucleotides	University of Pennsylvannia	Phase 1 - In Advanced Hematological Malignancies	NCT00780052

## Wnt signaling

The Wnt glycoproteins are a family of growth factors, important for developmental hematopoiesis and highly expressed within the BM niche. Wnt proteins signal through at least three intracellular-signaling pathways, however signaling via the canonical pathway is the best characterised in the hematopoietic system. To date, several members of the Wnt family (including Wnt-1, 3a, 5a and 10b), along with downstream components of the signaling pathway have been shown to stimulate proliferation and self-renewal of HSCs and are involved in their long term maintenance [37,38]. Aberrant activation of the Wnt/β-catenin pathway has been linked to promoting myeloid leukemia, with increased levels of active β-catenin and components of the Wnt pathway linked to AML [39], and progressive CML [40], suggesting that Wnt pathway activation is important for modulating leukemic hematopoiesis. Canonical Wnt signaling is initiated by the binding of a Wnt protein to its receptor, frizzled (Fz) family, and a co-receptor of the low-density-lipoprotein-receptor-related-protein family (LRP5 or LRP6) [41]. The central player in the canonical pathway is β-catenin. In the absence of Wnt signaling, β-catenin is present in a cytoplasmic ‘destruction complex’ and is continuously phosphorylated by casein kinase 1 (CK1) and GSK3β. This creates a recognition motif for an E3-ubiquitin-ligase complex that contains β−transducin-repeat-containing protein (β-TRCP), which tags the protein for proteasomal degradation. Wnt signaling inactivates GSK3β through interaction with the protein dishevelled (Dvl); Dvl is recruited to the cell membrane, allowing dissociation of GSK3β from the destruction complex and un-phosphorylated β−catenin to accumulate and translocate to the nucleus. A recently proposed modification to this pathway suggests that Wnt ligands only stop the ubiquitylation of β-catenin in preformed complexes, where phosphorylated substrate still remains bound [41]. This allows non phosphorylated substrate to accumulate, β-catenin then displaces co-repressors belonging to the groucho-related gene family (GRG) bound to TCF/LEF transcription factors, resulting in the induction of target genes such as *c-myc*, *c-jun* and *cyclinD1*[42]. Additional receptors for Wnt ligands include the single pass orphan receptor tyrosine kinases, ROR 1 & 2 involved in Wnt5a signaling and the atypical receptor tyrosine kinase, Ryk involved in Wnt3a signaling. Emerging evidence indicates that non-canonical Wnt signaling, especially via Wnt5a plays, an important role in modulating hematopoiesis. This pathway is involved in survival and the stabilization of intracellular signaling through calcium-mediated mechanisms. Non-canonical Wnt proteins bind Fz receptors which interact with the ROR family of heteromeric G proteins to activate phospholipase C, which in turn, generates diacylglycerol and inositol-phosphate 3 (IP3) and intracellular Ca^2+^ levels increase. This results in protein kinase C (PKC) and calmodulin-dependent kinase II (CamKII) activation [43]. Interestingly, non-canonical signaling has been shown to exert an antagonistic effect on canonical signaling, with Wnt5a promoting GSK3β independent degradation of β-catenin [44] and competing with Wnt3a for binding to the receptor complex [45]. Wnt signaling although recognised as a key pathway in modulating HSCs behaviour is largely dispensable for steady-state haematopoiesis, whereas increasing evidence supports a role for this pathway in myeloid malignancies, especially LSC maintenance. The Wnt pathway has been shown to be important for both CML and AML LSC self-renewal. In models of HoxA9/Meis1a or MLLAF9 induced AML, it has been shown that Wnt pathway components, such as Fzd4, 6, cyclinD2 and Frzb are unregulated in LSCs. It has also been demonstrated that β-catenin promotes AML, and that it is essential for disease initiation from LSCs and granulocyte-macrophage progenitor (GMP) cells. Wnt signaling is not active in normal GMPs, and this may partially account for their acquisition of stem-like properties [46,47]. Similarly, in CML, it has been demonstrated that GMPs with stem cell-like properties displayed higher levels of β-catenin and differential regulation of 16 Wnt pathway associated genes [48]. Consistent with a role in self-renewal, several positive regulators of the pathway were shown to be up-regulated, including CK1 and LRP6. Conversely, the negative regulator GSK3β was down-regulated. Further studies of murine models of CML have shown that decreasing levels of the Wnt pathway effector β−catenin in the hematopoietic compartment reduces CML stem cell self-renewal. As a consequence, these cells have an impaired ability to induce disease in their adoptive hosts. It is worth noting, that ALL progression was unaffected in this model [49]. In other CML murine models, Wnt signaling through β-catenin has been shown to play an important role in disease establishment and maintenance [49] with LSCs relying on β-catenin signaling for their survival following tyrosine kinase inhibitor (TKI) treatment [50] which can be prevented by combination targeting [26]. These studies indicate that the Wnt/ β-catenin pathway contributes to CML LSC survival especially following TKI treatment, highlighting its importance as a therapeutic target. The Wnt/GSK pathway, although potentially important in cancers, is currently not the subject of clinical trials due to a lack of clinical grade small molecule inhibitors [51]. However, given the emerging evidence for β-catenin in LSC persistence, this pathway offers an attractive therapeutic window for targeted therapies.

## Notch signaling

Interest in the role of Notch signaling in hematopoiesis arose following the discovery that Notch mutations occur at a high frequency in T-ALL [52]. High levels of the ligands that induce Notch signaling are present on the membrane of stromal cells, with Delta 1 & 4 and Jagged 1 & 2 expressed in the thymus, and Jagged 1 in the BM stroma [53,54]. Notch receptors are expressed by hematopoietic progenitor cells and by immature thymocytes (predominantly Notch 1 and Notch 3) [55,56]. Signaling is initiated when the large extracellular domain of the Notch receptor binds a membrane-bound ligand on a neighbouring cell. The glycosylation status of the Notch receptor is modified by Fringe proteins (radical, maniac and lunatic), and these post-translational modifications are important for ligand binding. When Notch interacts with a ligand, proteolytic cleavage is initiated at two sites; the first occurring externally to the transmembrane region and mediated by the action of ADAM metalloproteases. The second cleavage occurs within the transmembrane domain and is mediated by a multiprotein protease complex known as γ- secretase. This releases intracellular Notch (ICN), which translocates to the nucleus and binds to the nuclear transcription factor CSL. Binding induces the dislocation of repressors such as Mint and Nrap, and allows recruitment of co-activators, such as Mastermind (Mam1), resulting in the transcription of Notch target genes. The best known target genes encode Hairy-Enhancer of Split (*Hes)* 1 and *Hes5*, Hes-related repressor protein (*Herp*), *NRARP* and *Notch1*[57]. Although Notch signaling has been implicated in the maintenance of HSC, and is thought to cross-talk with Wnt signaling in the BM niche to regulate self-renewal capacity [58], its role in LSCs is still not fully defined, however various reports suggest it can suppress differentiation and augment the immature cell phenotype in myeloid leukemias. Consistent with this is the finding that Notch signaling is down-regulated in AML, and activation of it, targets LSCs, inducing apoptosis, and promoting differentiation to dendritic cells and macrophages [59,60]. Down-regulation of the pathway can also cooperate with loss of Tet2 to induce an AML-like disease in mice [59]. These studies demonstrate a potential therapeutic window using Notch receptor agonists to target AML. Down-regulation of the pathway has also been reported in CMML, with inhibition of Notch signaling resulting in an accumulation of aberrant myeloid progenitors and a CMML phenotype in mice [61]. In addition *Hes1* is highly expressed in blast-crisis CML and retroviral co-expression of *Hes1* with *BCR-ABL* in a murine model led to an aggressive acute leukemia [62].

To date, only clinical trials of the γ-secretase inhibitor (GSI) MK-0752 in hematopoietic malignancies have been performed in T-ALL patients (see Table 1); however a number of studies are currently recruiting in solid tumours, in particular breast cancer, where GSIs may play a role in reversing resistance to hormone therapies [63].

## Hedgehog signaling

Hedgehog (Hh) signaling plays an important role in developmental hematopoiesis, promoting HSC self-renewal and expansion to establish definitive hematopoiesis. However its role in adult hematopoiesis has been controversial with mouse genetic studies indicating that the pathway is dispensable for steady state hematopoiesis [64]. These findings may provide a therapeutic window that can be exploited in haematological malignancies as abnormal Hh signaling has been associated with AML and CML [34,65-68]. Hh ligands, Sonic (SHh), Desert (DHh) and Indian (IHh) are produced in the BM niche where they bind to their receptors patched (PTCH) 1 and 2, which releases PTCH inhibition of smoothened (SMO). SMO is a membrane protein related to G protein-coupled receptors, which when activated enhances nuclear translocation of the GLI family of zinc finger transcription factors, GLI1, GLI2, and GLI3. While GLI3 is predominantly a transcriptional repressor, GLI2 exists in both a full length active form and a truncated repressor form. Activated SMO alters the balance between these forms. In the absence of Hh signaling, SMO is inhibited, resulting in GLI2 and GLI3 being retained in the cytoplasm by a protein complex including the inhibitory molecule Suppressor of Fused (SUFU). This results in the phosphorylation of GLI2 and GLI3 at multiple sites by PKA, GSK3β and CKI, targeting them for proteasomal degradation to generate truncated repressor forms. In the presence of Hh ligand, SMO is enriched at the plasma membrane, GLI2 and GLI3 phosphorylation is prevented and full length active forms translocate to the nucleus [69]. This activates the transcription of downstream targets that include both positive (*GLI1*) and negative (*PTCH1/2*) -regulatory elements of the pathways as well as target genes such as the ATP-binding cassette (ABC) transporter family members; P-glycoprotein (*Pgp*), multi-drug resistance protein-1 (*MDR1*) and breast cancer resistance protein (*BCRP; ABCG2*) [70] as well as the anti-apoptotic gene *BCL-2*, whose transcription is directly regulated by GLI1 and GLI2 [71]. Several lines of evidence demonstrate the importance of the Hh pathway in the maintenance of LSCs in CML. The use of mouse models has demonstrated that retrovirally delivered BCR-ABL can initiate a CML-like disease that is stem cell dependant, Dierks and co-workers demonstrated that the Hh target genes Gli1 and Ptch were up-regulated in the LSCs compared to HSCs. A finding that was confirmed in CD34^+^ cells from CML patients relative to CD34^+^ cells from healthy controls [66]. In addition, SMO was up-regulated at the protein level, in all BCR-ABL positive cells in both the mouse model and human CML samples. Furthermore, inhibition of the Hh pathway by inactivating SMO with KAAD-cyclopamide caused a reduction in the self-renewal capacity of BCR-ABL positive LSCs. Further confirmation of the role of the Hh pathway comes from a murine model where SMO is deleted in the hematopoietic compartment during embryogenesis. In this model, BCR-ABL transduced BM cells showed lower LSC numbers and a resultant impairment in their ability to initiate CML-like disease compared to controls [66].

Inhibitors that antagonise SMO are currently being investigated in a number of trials, as outlined in Table 1.

## Transforming growth factor beta (TGFβ ) superfamily signaling

This family of ligands include TGFβ, the activins and the bone morphogenetic proteins (BMPs), which regulate an extensive array of fundamental processes during development and postnatally through the Smad signaling pathway. Ligand binding activates the serine/threonine kinase receptors to phosphorylate receptor activated Smads (R-Smads). TGFβ and activin signaling activate R-Smad 2 and 3, whereas BMP signals are transduced through R-Smads 1, 5 and 8. These associate with the common partner Smad 4 (Co-Smad 4), creating a complex which translocates to the nucleus to facilitate target gene transcription. Activation is prevented through inhibitory Smads (I-Smads) 6 & 7 [72]. TGFβ has potent anti-proliferative properties and is thought to be a key modulator of HSC quiescence. In addition, TGFβ plays a principle role in immune cell homeostasis and function with, mice developing a lethal inflammatory disorder when TGFβ1 or its receptors are knocked out [73,74]. *TGFβ1*-null mice also displayed enhanced myelopoiesis suggesting that TGFβ1 acts as a negative regulator of myelopoiesis [73]. BMPs have been implicated as key regulators of hematopoietic development in a variety of species, however their role in steady state adult hematopoiesis is unclear. Evidence suggests that BMPs and Hh cross-talk within the BM niche and play a role in controlling HSC numbers [75]. The TGFβ pathway has been implicated in the maintenance of LSCs in CML via Akt activation [76]. It has also been shown that in AML, TGFβ/BMP2 signaling can inhibit the re-plating potential of cells transformed by HoxA9 deregulation. This is achieved via the binding of Smad 4, which blocks the ability of HoxA9 and its fusion protein Nup98-HoxA9 to target DNA sequences [77].

## Hox genes

Another important group of self-renewal related genes are those of the Hox cluster. Wnt and BMP signaling converge to regulate the Cdx family of homeobox transcription factors, master regulators of Hox gene expression [78,79]. Consensus binding sites for the three Cdx homologues Cdx1, Cdx2, and Cdx4 are present in the promoters of multiple Hox genes [80,81]. Substantial evidence has now linked aberrant expression of Hox genes to the pathogenesis of myeloid malignancies, especially the self-renewal of AML and CML stem cells [82,83]. It is particularly noteworthy, that the majority of human AML samples display deregulation of Hox gene expression. This may in part be due to the aberrant expression of Cdx2, which is up-regulated in 90% of AML samples, but not expressed in normal adult hematopoietic tissue [84]. In AML, Cdx2 activation also results in the silencing of the transcription factor KLF4 which acts as a tumor suppressor in the myeloid compartment. Modulation of Cdx2 activity using the PPARγ agonist prostaglandin J2, can restore KLF4 levels, reduce human LTC-IC frequency (an *in vitro* surrogate marker of stem cell activity) and induce apoptosis in a murine model, making Cdx2 an attractive therapeutic target [85]. Fusions of the HoxA9 or HoxD13 genes with NUP98, a gene that encodes a component of the nuclear pore complex, have been described in AML, and recapitulates AML in murine models of disease [86,87]. Over-expression of individual Hox family members, including HoxB3 [88], HoxB8 [89], or HoxA10 [90], by retroviral expression or retroviral insertion mutagenesis also generates AML in murine models. In addition, up-regulation of specific Hox genes, such as HoxB4 or HoxA9, is associated with expansion of the HSC compartment *in vitro* and *in vivo* and results in enhanced competitive repopulating activity in murine transplantation experiments [91,92]. Cdx4, has been shown to enhance the replating potential of HSCs [92]. The targets of Cdx4 appear to be HoxA6, A7, A9, B4, B6, B8 and C6. HoxA9, for example, has been shown to be required for self-renewal and its expression correlates with a poor prognosis in CML [93]. The co-activator Meis1a is required for Cdx4 induced leukemia initiation. Meis1 also acts as a cofactor for HoxA7 and A9 in the generation of myeloid leukemias [92,94]. The HoxA9/Meis1 transcriptional regulation complex also up-regulates c-Myb, a current target for a phase 1 clinical trial, inducing a self-renewal transcriptional programme that overlaps with the embryonic c-Myc programme [95]. This pathway can also be induced by various chimeric MLL histone methyltransferase oncoproteins. The methyltransferases themselves may be good potential therapeutic targets as hDOT1L has been shown to maintain Hox gene expression by MLL-AF10, MLL-ENL and CALM-AF10 fusions [96]. Furthermore, recent small molecule inhibitors of DOT1L such as EPZ00477, have been developed, and shown to decrease MLL fusion protein target gene expression, induce cell cycle arrest and increase the expression of the myeloid differentiation marker CD14 in MOLM-13 cells [97,98]. Efficacious concentrations of this compound are also well tolerated by mice, making it an attractive prospect for clinical use [97].

## Cross-talk between signaling pathways

There are several potential points of cross-talk in malignant myeloid hematopoiesis. These include cross-talk with the stroma of the stem cell niche, interaction with normal HSCs and the immune system, and integration of intracellular signaling pathways in the LSC. Strategies that disrupt the LSC-stroma interaction could include the use of mobilising cytokines such as granulocyte-colony stimulating factor or agents that affect the homing and retention of LSCs in the stem cell niche. This would remove LSCs from a self-renewal supporting niche and potentially predispose them to differentiate. Such potential targets that have shown *in vivo* promise include the α4β1 integrin receptor VLA4, the CXCL12 receptor CXCR4 and the osteopontin receptor CD44 [99-103].

As highlighted above, certain intracellular signaling molecules impact on more than one pathway, indicative that these pathways converge to maintain self-renewal potential. One study reports on the role of STAT3 in cross-talk between the Wnt and Hh pathways [104]. The authors propose that paracrine SHh activates STAT3, which then turns on the expression of autocrine SHh and Wnt3a in CML cells, making the cells less dependent on the BM for these important self-renewal factors. In the BM niche, this increased expression of Wnt3a by the LSCs may also lead to an increase in Notch ligand production by stromal cells, providing further self-renewal signals. A role for STAT3 in up-regulating Wnt3a inducible genes (*Lef1* and *CyclinD1*) has also been suggested, however whether these effects are due to the transcription factor activity of STAT3, or its ability to interact with and modify the activity of other signaling proteins needs to be verified. The authors also report a small increase in the SHh pathway inhibitor PTCH1 following Wnt3a stimulation of CML cells, suggesting that Wnt signaling may play a negative regulatory role on the SHh pathway [104]. There is also a potential link between the HoxA10 down-regulation noted in this study and increased Wnt signaling as the negative Wnt pathway inhibitor protein dickkopf-1 requires HoxA10 for its expression [105]. A number of studies suggest that the regulation of β-catenin stability may be an important event in the regulation of LSC growth and self-renewal pathways. It has been suggested for example, that FoxO in response to ROS, may bind to β-catenin and stabilise it, causing enhanced signaling. It is also possible that FoxOs can compete with β−catenin for the limited cellular pools of TCF [106]. Other studies have also shown that E and N-cadherin interactions may also sequester β-catenin levels in HSCs, and down modulate the expression of Wnt targets of self-renewal, like Myc. This interaction can be perturbed by the transmembrane metalloproteinase ADAM10, which can cleave N-cadherin and allow β-catenin to access the Wnt signaling pathway [107]. This would make ADAM10 a potential therapeutic target that could modify Wnt signaling in both stromal and LSCs. β-catenin can also be stabilised via CD27 ligation. This is through an interaction with TRAF2 and NCK interacting kinase [108]. This complex binds to and transactivates the TCF/LEF complex.

FoxOs act as sensors of ROS, and effectors of the TGF and Notch pathway where they promote self-renewal [35]. However, too much ROS can kill LSCs, and the balance of ROS appears to be regulated in part through HoxB4 and Prdm16 mediated up-regulation of glutathione peroxidase 3, with high levels of expression favouring LSC maintenance and an aggressive AML phenotype [109]. In AML low levels of Akt activity has been associated with elevated FoxOs and enhanced maintenance of LSC’s whereas depletion of FoxO3 results in increased differentiation and apoptosis. Inhibition of FoxO either directly or via increased Akt activation can also result in enhanced JNK/c-Jun signaling which suppresses apoptosis of AML cells highlighting the importance of these pathways in sustaining the function and immature status of LSCs [110].

The activation of Akt inhibits the activity of GSK3β, potentiating Wnt and Hh signaling pathways as described above. In addition, in frame splice mutants of the GSK3β kinase domain increase β-catenin expression resulting in enhanced serial engraftment of blast-crisis CML progenitors into immunocompromised mice [111]. Furthermore, GSK3β may be required for the inhibition of Notch signaling, and the suppression of c-Myb expression [112]. As well as its negative effects on LSC self-renewal, in some instances evidence suggests that GSK3β activation can promote the proliferation/maintenance of LSCs. For example, MLL histone methyltransferase oncoproteins have been shown to be dependent on the GSK3β mediated activation of a variety of Hox/Meis1 target genes for their proliferative effects on LSCs [113]. Clearly, the function of GSK3β in LSC biology is context dependant, and therapeutic intervention will need to be sensitive to the particular molecular lesions and self-renewal pathways involved in any particular myeloid malignancy.

## Targeting self-renewal pathways in LSC

Many small molecule inhibitors have been developed which are capable of targeting different molecules within self-renewal pathways. These agents are at different stages of pre-clinical and clinical development; indeed some well known drugs, e.g. non-steroidal anti-inflammatory drugs (NSAIDs) have effects on self-renewal. Given that various pathways can cross-talk, or amplify the input signals the LSC receives, combined targeting of different pathways may prove to be a fruitful area of future investigation. Table 1 indicates those pathways where inhibitors have been developed and also identifies relevant ongoing or recently completed clinical trials in hematological malignancies using these agents.

## Conclusions

Self-renewal pathways are currently an area of major study in myeloid malignancies. There is increasing evidence that the development of novel clinical agents which specifically target LSCs is essential to cure these stem-cell derived malignancies. Thus, understanding the aberrant expression of self-renewal pathways and cross-talk between these pathways in LSCs is essential for the development of novel treatment strategies. It is highly likely that a successful strategy for curing leukemia will consist of a targeted LSC therapy in combination with standard chemotherapy agents (e.g. anthracyclines, cytosine arabinoside, fludarabine) in AML and TKIs in CML to eradicate the LSCs and kill the bulk leukemia blast cells, respectively. The timing of administration of different agents is likely to be critical and will require careful pre-clinical studies to ensure successful clinical trials. One possible approach would be induction therapy with standard chemotherapy in AML or TKIs in CML to reduce the bulk leukemia cells followed by LSC-directed therapy when tumor burden is low, i.e. minimal residual disease (MRD) has been achieved, to eliminate LSCs. For this approach to be successful further work needs to be performed in a number of areas including: (a) the development of more clinical grade self-renewal pathway modulators; (b) the development of sensitive and robust tests to monitor MRD (e.g. quantitative RT-PCR for *BCR-ABL* in CML); and (c) the development of assays to allow measurement of the rare LSC population size and functionality. When successfully achieved in myeloid leukemias, these approaches can then be adopted in other stem-cell driven malignancies.

## Competing interest

The authors declare no competing financial interests.

## Authors’ contributions

HW coordinated the review, wrote the sections of normal hematopoiesis and self-renewal pathways and compiled the figure; WS wrote the section on myeloid malignancies; MC researched the table and wrote the conclusion. All authors read and approved the final manuscript.
